# AA Amyloidosis: A Contemporary View

**DOI:** 10.1007/s11926-024-01147-8

**Published:** 2024-04-03

**Authors:** Safak Mirioglu, Omer Uludag, Ozge Hurdogan, Gizem Kumru, Ilay Berke, Stavros A. Doumas, Eleni Frangou, Ahmet Gul

**Affiliations:** 1 Division of Nephrology, Istanbul Faculty of Medicine, Istanbul University, Istanbul, Turkey; 2 Department of Immunology, Aziz Sancar Institute of Experimental Medicine, Istanbul University, Istanbul, Turkey; 3 Division of Rheumatology, Istanbul Faculty of Medicine, Istanbul University, Istanbul, Turkey; 4 Department of Pathology, Istanbul Faculty of Medicine, Istanbul University, Istanbul, Turkey; 5 Division of Nephrology, Ankara University Faculty of Medicine, Ankara, Turkey; 6 Division of Nephrology, Marmara University School of Medicine, Istanbul, Turkey; 7 Department of Medicine, MedStar Georgetown University Hospital, Washington, DC USA; 8 Department of Nephrology, Limassol General Hospital, State Health Services Organization, Limassol, Cyprus; 9 University of Nicosia Medical School, Nicosia, Cyprus

**Keywords:** Amyloidosis, Arthritis, Autoinflammatory diseases, Chronic infection, Chronic inflammation, Serum amyloid A

## Abstract

**Purpose of Review:**

Amyloid A (AA) amyloidosis is an organ- or life-threatening complication of chronic inflammatory disorders. Here, we review the epidemiology, causes, pathogenesis, clinical features, and diagnostic and therapeutic strategies of AA amyloidosis.

**Recent Findings:**

The incidence of AA amyloidosis has declined due to better treatment of the underlying diseases. Histopathological examination is the gold standard of diagnosis, but magnetic resonance imaging can be used to detect cardiac involvement. There is yet no treatment option for the clearance of amyloid fibril deposits; therefore, the management strategy primarily aims to reduce serum amyloid A protein. Anti-inflammatory biologic agents have drastically expanded our therapeutic armamentarium. Kidney transplantation is preferred in patients with kidney failure, and the recurrence of amyloidosis in the allograft has become rare as transplant recipients have started to benefit from the new agents.

**Summary:**

The management of AA amyloidosis has been considerably changed over the recent years due to the novel therapeutic options aiming to control inflammatory activity. New agents capable of clearing amyloid deposits from the tissues are still needed.

**Supplementary Information:**

The online version contains supplementary material available at 10.1007/s11926-024-01147-8.

## Introduction

Disorders that create sustained inflammatory responses can result in extracellular deposition of amyloid A (AA) fibrils in various organs. These fibrils are derived from an acute-phase reactant, serum AA (SAA) protein, through a long process including cleavage, misfolding, and aggregation into an insoluble beta-sheet form [1]. The incidence of AA amyloidosis has been low in recent years especially in developed countries due to declining rates of chronic infections and better treatment strategies for inflammatory conditions including autoimmune and autoinflammatory diseases. However, it still continues to be a significant cause of morbidity and mortality if left untreated or undertreated. Herein, we will review the causes, pathogenesis, and clinical features with an emphasis on histological diagnosis as well as conventional and novel treatment strategies of AA amyloidosis. As the kidney is the major affected organ and the damage in kidneys usually defines the prognosis of AA amyloidosis [2], we will devote a separate section to various aspects of patients treated with kidney replacement therapies.

## Epidemiology and Causes

The incidence of AA amyloidosis has been reported as one to two cases per million person-years [3, 4], yet these rates may be higher in resource-limited settings. As the incidence is in decline, its frequency among all forms of amyloidosis is being replaced by other types such as immunoglobulin light chain (AL) and transthyretin amyloidosis [2], and a recent proteomics study involving 16,175 samples revealed that only 2.9% of all cases suffered from AA amyloidosis [5, 6••]. Median age at diagnosis has been historically around 50 years, but it has recently been reported as high as 70 [7, 8]. Males are slightly more affected [8].

Even though any disorder causing sustained inflammation may increase the risk of AA amyloidosis, chronic infections and inflammatory arthritis are the commonest causes [2]. Primary immunodeficiencies leading to infections and immune dysregulation may create a state of chronic inflammation, and in particular, delays in diagnosis and therapeutic interventions were associated with the development of AA amyloidosis [9]. Monogenic periodic fever syndromes also constitute a significant risk for amyloidosis, and familial Mediterranean fever (FMF) is a prevalent cause for people originating from the Mediterranean basin [10•]. A substantial proportion of cases (up to 20%) appear to develop AA amyloidosis in the absence of overt inflammation and thus labeled idiopathic [11]. Obesity was suggested as a cause or susceptibility factor [5, 12], but yet undefined genetic and/or environmental causes might be involved in the pathogenesis of both obese and idiopathic cases [2]. The causes of AA amyloidosis are detailed in Table 1.

**Table 1:** Causes of AA amyloidosis

Chronic infections	Chronic inflammatory disorders
* Bacterial infections*	* Inflammatory arthritis*
Tuberculosis	Rheumatoid arthritis
Leprosy	Spondyloarthritis
Whipple disease	Psoriatic arthritis
Osteomyelitis	Juvenile idiopathic arthritis
Chronic pyelonephritis	Adult onset Still disease
Chronic cutaneous ulcers	Gout
Abdominal infection	
Bronchiectasis	* Vasculitis*
Infections related to injected drug use	Giant cell arteritis
	Takayasu arteritis
* Other infections*	Polyarteritis nodosa
Aspergillosis	Behçet’s disease
Hepatitis B	
HIV	* Autoinflammatory diseases*
Immunodeficiencies	FMF
CVID	NLRP3-AID (CAPS)
Hypo/agammaglobulinemia	TRAPS
X-linked agammaglobulinemia	MKD (HIDS)
Cyclic neutropenia	
Hyper IgM syndrome	* Other rheumatic diseases*
Chronic granulomatous disease	SLE
Hematologic diseases	Mixed connective tissue disease
Hodgkin’s disease	Sjögren syndrome
Non-Hodgkin lymphoma	Polymyalgia rheumatica
Hairy cell leukemia	
Waldenström macroglobulinemia	* Inflammatory bowel diseases*
Multiple myeloma	Crohn’s disease
Schnitzler syndrome	Ulcerative colitis
Castleman’s disease	
Solid tumors	* Others*
Renal cell carcinoma	Hidradenitis suppurativa
Lung cancer	Epidermolysis bullosa
Basal cell carcinoma	Sarcoidosis
Gastrointestinal stromal tumor	IgG4-related disease
Hepatocellular adenoma	SAPHO syndrome
Mesothelioma	
Sarcoma	
Urogenital cancers	
Cancer therapy (immune checkpoint inhibitors)	

Supplementary Table 1 is the version with references

*CAPS* cryopyrin-associated periodic syndrome, *CVID* common variable immunodeficiency, *FMF* familial Mediterranean fever, *HIDS* hyper IgD syndrome, *HIV* human immunodeficiency virus, *MKD* mevalonate kinase deficiency, *NLRP3-AID* NLRP3-associated autoinflammatory disorder, *SAPHO* synovitis, acne, pustulosis, hyperostosis and osteitis, *SLE* systemic lupus erythematosus, *TRAPS* tumor necrosis factor receptor–associated periodic syndrome

## Pathogenesis

Pathogenesis of AA amyloidosis revolves around a cascade of events triggered by sustained inflammation and increased production of SAA, cleavage of the signal peptide, aberrant aggregation, SAA-derived fibril formation, and deposition of amyloid fibrils [13]. Amyloid formation typically involves misfolding of soluble precursor proteins into a beta-sheet structure, and the monomeric protein then aggregates into fibril conformations that are insoluble and show resistance to proteolytic enzymes [14]. Thirty-six different monomeric human precursor proteins like SAA have been recognized so far with the potential to form amyloid fibrils, which then cause systemic or localized amyloidosis [15].

SAA is an apolipoprotein of high-density lipoprotein synthesized especially by hepatocytes as an acute-phase reactant, primarily under the influence of pro-inflammatory cytokines such as interleukin-1 (IL-1), IL-6, and tumor necrosis factor-alpha (TNF-α) [16]. SAA is involved in lipid transfer and immune regulation during acute-phase responses including cytokine induction, matrix metalloproteinase (MMP) stimulation, phagocyte migration, chemoattraction, and bacterial opsonization [16, 17]. Also, it has a role in the atherosclerosis [18] through an interaction between lipid homeostasis and inflammatory cells and contributes to chronic injury in arthritis and tumor metastases [19, 20]. Table 2 summarizes several important factors that contribute to the amyloidogenic potential of SAA in addition to its physiologic functions.

**Table 2:** The factors contributing to the amyloidogenic potential of SAA in addition to its physiologic functions

SAA level increases to a critical concentration for a prolonged period [13, 21]
The 122-amino acid-long SAA pre-protein is cleaved to a 76-amino acid AA protein by different MMPs. The variations in the SAA cleavage site contribute to its misfolding and aggregation [22, 23]
Polymorphisms within the *SAA1* gene can impact the expression, structure, and function of the SAA protein, thereby influencing the susceptibility and severity of AA amyloidosis [24]
Physiological factors such as acidic pH, high temperature, and addition of heparin may increase resistance to proteolysis of SAA oligomers and promote accumulation [25, 26]
Morphological and structural differences between amyloid fibrils may identify their amyloidogenic property [27, 28]
Molecules other than amyloid fibrils such as SAP component, heparan sulfate, and apolipoproteins play a role in amyloid fibril formation and protection [29]
Although the tissue damage mechanisms of amyloidosis are not fully known, direct cytotoxicity or disruption of tissue structure due to aberrant amyloid accumulation may contribute to its pathogenicity [29, 30]

*MMP* matrix metalloproteinase, *SAA* serum amyloid A, *SAP* serum amyloid P

Human SAA proteins are encoded by four *SAA* genes on chromosome 11p15.1 [21]. *SAA1* and *SAA2* are both acute-phase proteins and associated with AA amyloidosis. *SAA3* is a pseudogene, and *SAA4* is a constitutively expressed non-acute phase protein. *SAA1*, which predominates AA deposits, has 5 polymorphic alleles (*SAA1.1–1.5*) with differing amyloidogenic potential among them. Homozygosity for *SAA1.1* in the European population [22, 23] and *SAA1.3* in the Japanese population [25] increases the risk of amyloidosis, which can be partially explained by the increased susceptibility of SAA1.1 allele to degradation by MMP than SAA1.5 [26]. Inducible expression of *SAA1* and *SAA2* by proinflammatory cytokines during acute-phase response impacts the serum concentration of SAA [21]. Moreover, certain single nucleotide variations within the *SAA* genes can also affect the production and stability of SAA isoforms [31], and genotype–phenotype associations have been well described in patients with periodic fever syndromes [32]. In addition to the homozygosity for the most penetrant *MEFV* gene variant M694V, which is associated with a severe disease course [8], homozygosity for *SAA1.1* allele contributes to the risk of amyloidosis in FMF patients [33]. Recently, a family with AA amyloidosis due to a genetic mutation in the promoter region of the *SAA1* gene has also been reported [24]. In a multicenter study, patients’ country of recruitment [34] has been identified as a risk factor for kidney amyloidosis, more important than the *MEFV* genotype and disease duration in FMF, which suggests that ethnicity and environmental factors may play an additional role.

## Diagnosis

### Clinical Features

AA amyloidosis manifests in multiple organs such as kidney, liver, gastrointestinal tract, peripheral nerves, heart, blood vessels, lungs, skin, and soft tissue. The expression of the disease is contingent upon the specific organs involved. Nevertheless, the prevailing symptoms frequently involve the emergence of proteinuria and gradual reduction in kidney function, apparent in over 90% of patients upon their initial presentation [1]. Nephrotic syndrome is noted in more than half of the patients, with approximately 10% exhibiting kidney failure at the time of presentation [1, 35, 36]. The site of the protein accumulation in kidneys varies, influencing clinical manifestations and disease progression. Typically, the involvement of glomeruli precipitates nephrotic-range proteinuria and an accelerated decline in estimated glomerular filtration rate (eGFR) [37]. Conversely, localized amyloid deposition within tubulointerstitium tends to result in less pronounced proteinuria. Individuals with tubular amyloid accumulation might exhibit bland urinary sediment and minimal proteinuria but can demonstrate symptoms of distal tubular impairment such as nephrogenic diabetes insipidus. Notably, crescentic glomerulonephritis, a rare event, may be ascribed to the rupture of the glomerular basement membrane due to amyloid deposition [38]. While the majority of cases follow an indolent course, a subset of individuals with FMF might undergo acute illness, marked by substantial proteinuria, elevated inflammatory markers, and rapid progression to kidney failure within weeks. These infrequent instances are named “amyloid storm,” which is thought to be precipitated by superimposed infections or other triggering events [39••].

Approximately 30% of cases have gastrointestinal involvement. The predominant symptom is diarrhea that is frequently unresponsive to standard treatment [11]. Other frequently encountered presenting symptoms include weight loss, abdominal pain, malabsorption, macroglossia, gastroesophageal reflux, esophageal dysmotility, gastric polyps, and episodes of upper and lower gastrointestinal bleeding [40]. Amyloid deposition in the myenteric plexus can potentially result in intestinal pseudo-obstruction [41]. While splenic and hepatic amyloid deposition is commonly detected in serum amyloid P (SAP) scintigraphy, its clinical relevance is limited [42]. Hepato- and splenomegaly commonly manifests during disease progression, whereas splenic rupture is exceptionally rare [1]. Elevated serum alkaline phosphatase (ALP) levels are noted in 5% of patients [1]. However, this may more accurately reflect underlying disease activity rather than amyloid deposition given that ALP functions as an acute-phase reactant. Incidences of increased serum aminotransferase and bilirubin levels have been seldomly reported, and liver failure is quite infrequent [43]. While cardiac infiltration is extensively documented in various amyloidosis types, it is a relatively rare cause of heart failure in AA amyloidosis [44]. Involvements of the thyroid, adrenals, and the nervous system might also be encountered [1, 11, 45].

### Histology

A biopsy from the involved tissue demonstrating amyloid deposits provides the definitive diagnosis. Amyloid deposits within the tissue typically show high affinity for Congo red stain, which gives a characteristic birefringence under polarized light. For the diagnosis of AA amyloidosis subgroup, immunohistochemical analysis with anti-AA antibody is required [46]. Since AA amyloidosis is clinically more limited compared to AL amyloidosis and mostly involves the kidney, a kidney biopsy is usually rendered for diagnosis. Gastrointestinal tract, liver, spleen, and rarely heart can also be the location of a biopsy [46]. Salivary gland biopsy and periumbilical subcutaneous fat aspiration, or retrospective analysis of prior biopsies with Congo red staining may help as less invasive approaches with an overall sensitivity ranging from 77 to 89% [47–49]. If clinical suspicion is significantly high and these approaches render negative results, a sample should be retrieved from the affected organ.

**Fig. 1:**
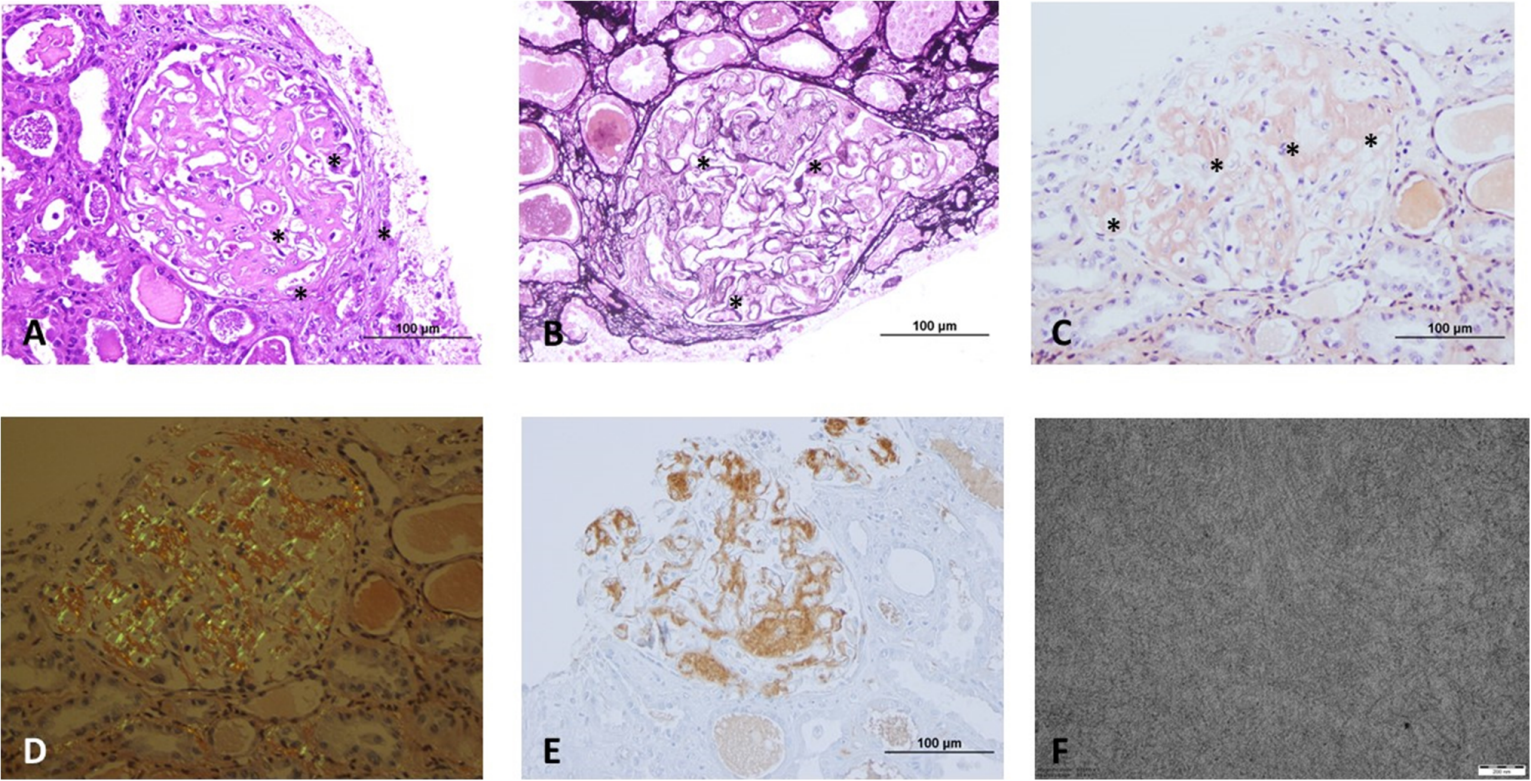
Kidney histology in a patient with AA amyloidosis. **A** Glomerulus with mesangial amorphous and eosinophilic deposits (hematoxylin and eosin, original magnification × 400). **B** Deposits not staining for silver methenamine (periodic Schiff-methenamine, original magnification × 400). **C** Congophilic glomerular deposits (Congo red, original magnification × 400). **D** Deposits showing apple green birefringence under polarized light (Congo red under polarized light, original magnification × 400). **E** Immunohistochemical reactivity for anti-AA antibody (original magnification × 400). **F** Randomly oriented, unbranched amyloid fibrils on electron microscopic examination (original magnification × 40,000). Deposits were marked with asterisks in panels **A**, **B** and **C**

The histopathological findings in the kidney of a patient with AA amyloidosis are shown in Fig. 1. Kidney deposits may be located in various compartments such as mesangium, glomerular basement membrane, arteries and arterioles, tubules, or interstitium [50]. It is typical for amyloid deposits to involve the mesangium first and the capillaries later, and if the entire glomerulus is involved with a nodular configuration, the histopathologic appearance may mimic light chain deposition disease, sclerosis, or diabetic nephropathy [51, 52]. Tubulointerstitial deposits might result in tubular atrophy and interstitial fibrosis [53]. The presence of amorphous hyaline deposits should raise a suspicion of amyloidosis which requires further examination with Congo red stain. These deposits typically show weak staining for periodic acid-Schiff and no staining for silver methenamine as the predominance of amyloid deposits reduces collagen deposition within the tissue [51]. Congo red stain should be routinely performed to rule out potential amyloid deposits since the deposits may be inconspicuous during the early courses of the disease and may lead to misdiagnosis of a podocytopathy in a patient with nephrotic-range proteinuria [54]. On electron microscopic examination, amyloid appears as rigid, randomly oriented, unbranched fibrils with a thickness of 8–12 nm in diameter. The fibril diameter is essential in differentiating amyloidosis from kidney diseases with fibrillary deposits, such as fibrillary glomerulonephritis with 15–20 nm and immunotactoid glomerulonephritis with 30–60 nm fibril diameters [55].

In systemic amyloidosis, the amyloid precursor protein must be specified to guide the patient management [56]. Immunohistochemical analysis for SAA protein is the most commonly used method to detect AA amyloidosis [56]. Alternatively, direct immunofluorescence for kappa and lambda light chains on fresh frozen tissue is a reliable method to detect amyloid deposits and rule out AL amyloidosis in kidney biopsies [56]. Since various amyloid subtypes may involve different glomerular parts, these features are not entirely specific, and subtyping still requires immunohistochemical methods with specific antibodies against precursor proteins [50].

The accuracy of immunohistochemical technique depends on the quality of antibodies and the experience of the pathologist; hence, it may fail to determine the amyloid subtype accurately [57]. A false positive result is a diagnostic pitfall in especially determining AA amyloidosis [58]. Immunoelectron microscopy may yield higher sensitivity and specificity, identifying the amyloid subtype in more than 99% of cases; however, it suffers from limited use and lack of global experience [48, 59]. In the last decade, laser microdissection/mass spectrometry (LD/MS) has been used as an alternative but expensive method for amyloid detection, where amyloid deposits are extracted from the tissue via laser microdissection and then processed for protein extraction and mass spectrometer analysis. Although immune methods are still currently used, newer methods are expected to provide a more accurate diagnostic evaluation, especially when the standard approaches fail [53, 54, 60].

### Imaging

Whole-body ^123^I-labeled SAP scintigraphy demonstrates a high sensitivity, reaching up to 90%, in diagnosing both AA and AL amyloidosis [61]. Additionally, it provides insights into the extent of organ involvement. Although it does not replace histological evaluation, SAP scintigraphy has substantially improved diagnostic precision and has been proven as a valuable tool in disease progression and monitoring therapy [62]. However, routine diagnostic application of SAP scintigraphy has a significant obstacle due to its very limited accessibility. It is exclusively performed in highly specialized centers, primarily owing to its high costs and technical intricacies. Moreover, the limited half-life of the isotope renders it impractical for the assessment of cardiac involvement [62]. Additionally, the capacity to evaluate intracerebral or peripheral nerve amyloidosis is impeded by the gradual infiltration of SAP into the nervous system [1].

There are several imaging modalities available for assessing cardiac amyloidosis. Two-dimensional Doppler echocardiography has been traditionally employed to illustrate the thickening of ventricular walls and valves, as well as to assess diastolic restrictive dysfunction associated with amyloid infiltration. Cardiac involvement is characterized by a mean left ventricular wall thickness exceeding 12 mm, excluding hypertension or other causes of left ventricular hypertrophy [63]. Cardiac magnetic resonance (CMR) imaging is gaining prominence in the swiftly evolving domain of cardiac investigations. Notably, late or diffusely distributed subendocardial gadolinium enhancement serves as a distinctive hallmark of amyloidosis in CMR [64]. Even though use of gadolinium in patients with low eGFR was traditionally discouraged, American College of Radiology group II and III agents have been reported to be very safe [65, 66]. While the specific role of CMR in tracking the progression or regression of amyloidosis remains undefined, the application of equilibrium CMR, previously validated for fibrosis, holds promise as a valuable tool for quantifying amyloid [67].

## Treatment

### General Principles and Supportive Measures

The management of AA amyloidosis has been drastically changed over the recent decades as novel therapeutic options have transformed the treatment of inflammatory conditions [36]. Historically, our options were restricted to medications that would nonspecifically target inflammation, such as colchicine, non-steroidal anti-inflammatory agents, glucocorticoids and even cyclophosphamide, with somewhat variable but potentially favorable results if started in a timely fashion [68–70]. As our understanding of the pathogenesis of inflammatory diseases progressed and our therapeutic armamentarium expanded with tailored potent anti-inflammatory therapies, there has been a paradigm shift in AA amyloidosis therapeutics.

The mainstay of treatment is targeted at managing the underlying pathogenic mechanisms by suppressing the cytokine-induced production of SAA protein by the liver. Notably, controlling inflammation is time-sensitive as delayed control of the inflammation cannot prevent the development of amyloid fibril deposits and can lead to disease progression [1]. Hence, treatment options are tailored accordingly, with infections treated promptly with antimicrobials, and rheumatological and genetic autoinflammatory conditions treated with the appropriate anti-inflammatory therapies. The monitoring of the treatment response is essential by the assessment of inflammatory symptoms as well as biochemical parameters with the measurements of SAA, as decreased levels have been consistently associated with the regression of amyloid deposition, improved organ function, and reduced mortality, or conversely, uncontrolled high SAA concentrations have been linked to progressive disease and increased mortality [1, 71].

Supportive measures are usually needed during the disease course, especially when the kidneys are affected. It is imperative to prevent further insults such as hypoperfusion or nephrotoxic drug use. In nephrotic syndrome or nephrotic-range proteinuria, angiotensin-converting enzyme inhibitors or angiotensin receptor blockers are the first choice of drugs with antiproteinuric properties, and patients should restrict dietary sodium to < 2 g/day to reduce edema [72]. Loop diuretics are generally required to manage hypervolemia, which should be combined with mechanistically different diuretics in resistant cases [72]. Hyperlipidemia, hypercoagulability, and symptoms originating from each affected organ should be treated according to the guidelines/recommendations.

In light of the above, we will review the different anti-inflammatory agents used in the treatment of AA amyloidosis and the recently investigated experimental therapies.

### Systemic Anti-inflammatory Agents

*Colchicine* has become the mainstay of FMF treatment after its efficacy in preventing attacks was demonstrated in 1970s [73]. It suppresses inflammasome activation and IL-1β production by inhibiting caspase-1 and causes secondary reductions in other pro-inflammatory cytokines like TNF-α and IL-6 [74]. In a study examining 960 patients with FMF without evidence of amyloidosis at baseline, the cumulative rate of proteinuria after 11 years was reported as 1.7% in colchicine-compliant patients while 48.9% in the non-compliants [75]. Additionally, proteinuria resolved in 5 of 86 (5.8%) patients with non-nephrotic range proteinuria and stabilized in 68 (79.1%). In another study evaluating 68 FMF patients with amyloidosis, deterioration in kidney function was associated with a baseline serum creatinine of > 1.5 mg/dl and colchicine dose of < 1.5 mg/day [76]. However, evidence for its efficacy in AA amyloidosis due to causes other than FMF is still quite limited [68, 77].

Studies evaluating *anti-TNF therapy* in AA amyloidosis predominantly include patients with rheumatoid arthritis and spondyloarthritis. In a retrospective series including 15 patients treated with anti-TNF, amyloidosis progressed in seven patients (46.7%), stabilized in five (33.3%), and regression of proteinuria occurred in three (20%) in 10 months [78]. Notably, in a multicenter study in which 36 patients with AA amyloidosis treated with anti-TNF therapy were followed prospectively for 5 years, > 50% decrease in proteinuria was observed in more than half of the patients [79]. Starting anti-TNF agents early in the disease course, especially when the baseline serum creatinine level was < 1.5 mg/dl, may improve the prognosis of the kidney disease [80]. Moreover, several reports showed improvement in AA amyloidosis with tocilizumab (*anti-IL-6 receptor* monoclonal antibody) treatment [81]. Notably, a retrospective analysis including 42 patients with AA amyloidosis demonstrated that tocilizumab was superior to anti-TNFs in terms of obtaining a decrease in SAA, improvement in kidney function, and suppression of the disease activity [82]. Anti-TNF agents are also used to treat inflammatory bowel diseases and improvement of AA amyloidosis in these patients has also been reported [83].

Considering the central role of IL-1 in the pathogenesis of autoinflammatory diseases, *IL-1 blockade* has become an important target. Efficacies of anakinra (a recombinant homolog of the human IL-1 receptor antagonist), rilonacept (an IL-1 receptor fusion protein), and canakinumab (fully human anti-IL-1β monoclonal antibody) in monogenic inflammasomopathies such as FMF, NLRP3-associated autoinflammatory disorder (formerly known as cryopyrin-associated periodic syndrome), TNF receptor-associated periodic syndrome (TRAPS), mevalonate kinase deficiency, and in other inflammasome-associated diseases with complex etiology such systemic onset juvenile idiopathic arthritis have been extensively shown in various studies [84••, 85–89, 90••, 91–93].

### Experimental Therapies

The advent of assays capable of defining the structure of the SAA has allowed us to understand better the biochemical basis of this protein folding disorder and explore the possibility of tailoring drugs aimed at protein misfolding prevention and promoting its removal [27, 94]. First significant attempt at exploring this was *eprodisate*, a molecule preventing SAA deposition by interacting with its polymerization. Initial results reported that eprodisate slowed the kidney function decline [95], which was not confirmed in a randomized control trial (NCT01215747) [96].

Further efforts have been attempted to deactivate the chaperone protein SAP which is responsible for facilitating the folding of amyloid proteins [97]. *Miridesap*, a serum SAP inhibitor, was initially investigated in a mixed amyloidosis population, followed by subsequent trials where it was administered in tandem with dezamizumab, a monoclonal antibody targeting SAP tissue depositions [98, 99•, 100]. These interventions appeared to remove the amyloid deposited in the organs of a small sample of patients, but the development was unfortunately discontinued due to fatal adverse effects during the clearance of amyloid deposits. Subsequently, a per os formulation of miridesap that achieves similar adequate blood levels as the parenteral formulation was reported, but no further investigation was pursued as arrhythmias were observed in the initial study [101].

The use of *anti-sense oligonucleotides* suppressing SAA production demonstrated a decreased amyloid organ deposition, yet its effects have not been studied in humans to date [102]. The development of new treatment options, which can effectively clear amyloid deposits from the tissues, is needed.

## Dialysis and Kidney Transplantation

Dialysis modalities and kidney transplantation are both suitable for patients suffering from AA amyloidosis and kidney failure [5]. Still, survival is poor among patients on dialysis, especially when cardiac involvement is prevalent [103]. Notably, the tendency to hypotension due to cardiovascular amyloid deposition and nephrotic syndrome might constitute a significant problem in these patients.

Even though kidney transplantation is the optimal treatment, 10-year patient and graft survival in patients with AA amyloidosis were reported as low as 62.3% and 56.4%, respectively [104]. However, contemporary series from the last years demonstrated higher patient and graft survival rates [105, 106••]. Recurrence is associated with a quite dismal prognosis [105], and it has recently become scarce due to better diagnosis and treatment of the underlying disorders [106••], but the diagnosis of subclinical recurrence in the graft should not be overlooked as many centers do not routinely perform protocol biopsies. Mortality after transplantation has been reported as high, mostly due to the involvement of the cardiovascular system [105, 107]. Therefore, a thorough cardiac assessment before transplantation has the utmost significance in patients with AA amyloidosis although its utility in the general transplant population is currently under debate.

Ant-TNF use in kidney transplant recipients (KTRs) has been associated with a better control of inflammation and remission of the underlying disease at the cost of increased rate of infections [108]. The results are expected as these agents are commonly used on top of maintenance immunosuppression. Notably, we recently evaluated 36 KTRs with FMF who showed resistance or inadequate response to colchicine and used anakinra or canakinumab in comparison with a propensity score-matched control group of KTRs [109••]. Anakinra and canakinumab were quite effective, culminating in longer graft survival and lower rejection rates but an increased number of deaths, which might have originated not only from infections but also the progression of the deposition of amyloid fibrils in the cardiovascular system. Colchicine has been reported to be safe in KTRs [105], but tacrolimus should be preferred over cyclosporine since the latter might increase the serum level or the effects of colchicine resulting in toxicity [110].

## Conclusions

AA amyloidosis is a serious complication of chronic inflammatory disorders and predominantly affects the kidneys. It has become quite rare due to the reduced rates of chronic infections and better treatment strategies for highly inflammatory autoimmune and autoinflammatory diseases. Diagnosis still relies on histology, and the therapeutic approach must aim to reduce SAA levels and keep in within normal limits. In addition to conventional therapies, biologic agents such as anti-TNFs and IL-1 and IL-6 antagonists have considerably expanded our therapeutic armamentarium. Kidney transplantation is preferred when amyloidosis results in progression to kidney failure. Recurrence after transplantation has also become scarce due to the aforementioned treatment options, but higher rates of infection and mortality might be of concern. Experimental therapies aiming to clear amyloid deposits from the tissues have not been successful so far.

### Supplementary Information

Below is the link to the electronic supplementary material.

Supplementary file1 (DOCX 20 KB)

## Data Availability

No datasets were generated or analysed during the current study.
