# Supplementary material for: AA Amyloidosis: A Contemporary View
Source: Curr Rheumatol Rep. 2024 Apr 3;26(7):248–59. doi: 10.1007/s11926-024-01147-8 (PMC11219434; doi:10.1007/s11926-024-01147-8)
Supplement: Supplementary file 1 — Supplementary file1 (DOCX 20 KB) [file 11926_2024_1147_MOESM1_ESM.docx]

**SUPPLEMENTARY DOCUMENT**

**Supplementary Table 1.** Causes of AA amyloidosis.

| **Chronic infections**  *Bacterial infections*  Tuberculosis [1]  Leprosy [2]  Whipple disease [3]  Osteomyelitis [1]  Chronic pyelonephritis [4]  Chronic cutaneous ulcers [5]  Abdominal infection [6]  Bronchiectasis [1]  Infections related to injected drug use [1]    *Other infections*  Aspergillosis [7]  Hepatitis B [8]  HIV [9] | **Chronic inflammatory disorders**  *Inflammatory arthritis*  Rheumatoid arthritis [1]  Spondyloarthritis [10]  Psoriatic arthritis [11]  Juvenile idiopathic arthritis [12]  Adult onset Still disease [13]  Gout [14]  *Vasculitis*  Giant cell arteritis [15]  Takayasu arteritis [16]  Polyarteritis nodosa [17]  Behçet’s disease [18]  *Autoinflammatory diseases*  FMF [19]  NLRP3-AID (CAPS) [12]  TRAPS [12]  MKD (HIDS) [12]  *Other rheumatic diseases*  SLE [20]  Mixed connective tissue disease [21]  Sjögren syndrome [22]  Polymyalgia rheumatica [15]  *Inflammatory bowel diseases*  Crohn’s disease [23]  Ulcerative colitis [23]  *Others*  Hidradenitis suppurativa [24]  Epidermolysis bullosa [25]  Sarcoidosis [26]  IgG4-related disease [27]  SAPHO syndrome [28] |
| --- | --- |
| **Immunodeficiencies**  CVID [29]  Hypo/agammaglobulinemia [29]  X-linked agammaglobulinemia [29]  Cyclic neutropenia [30]  Hyper IgM syndrome [29]  Chronic granulomatous disease [29] |  |
| **Hematologic diseases**  Hodgkin’s disease [31]  Non-Hodgkin lymphoma [1]  Hairy cell leukemia [32]  Waldenström macroglobulinemia [33]  Multiple myeloma [33]  Schnitzler syndrome [33]  Castleman’s disease [1] |  |
| **Solid tumors**  Renal cell carcinoma [34]  Lung cancer [34]  Basal cell carcinoma [34]  Gastrointestinal stromal tumor [34]  Hepatocellular adenoma [34]  Mesothelioma [1]  Sarcoma [34]  Urogenital cancers [34]  Cancer therapy (immune checkpoint inhibitors) [34] |  |

**Abbreviations:** CAPS: cryopyrin-associated periodic syndrome, CVID: common variable immunodeficiency, FMF: familial Mediterranean fever, HIDS: hyper IgD syndrome, HIV: human immunodeficiency virus, MKD: mevalonate kinase deficiency, NLRP3-AID: NLRP3-associated autoinflammatory disorder, SAPHO: synovitis, acne, pustulosis, hyperostosis and osteitis, SLE: systemic lupus erythematosus, TRAPS: tumor necrosis factor receptor-associated periodic syndrome.

**Supplementary References**

1. Lachmann HJ, Goodman HJ, Gilbertson JA, Gallimore JR, Sabin CA, Gillmore JD, et al. Natural history and outcome in systemic AA amyloidosis. N Engl J Med. 2007;356(23):2361-71. doi: 10.1056/NEJMoa070265.

2. Looi LM, Jayalakshim P, Lim KJ, Rajagopalan K. An immunohistochemical and morphological study of amyloidosis complicating leprosy in Malaysian patients. Ann Acad Med Singap. 1988;17(4):573-8.

3. Farr M, Morris C, Hollywell CA, Scott DL, Walton KW, Bacon PA. Amyloidosis in Whipple's arthritis. J R Soc Med. 1983;76(11):963-5. doi: 10.1177/014107688307601115.

4. Tanaka T, Naito T, Midori Y, Nosaka T, Takahashi K, Ofuji K, et al. Gastrointestinal AA amyloidosis secondary to chronic pyelonephritis presenting with refractory diarrhea and severe hypoalbuminemia. Clin J Gastroenterol. 2021;14(6):1642-8. doi: 10.1007/s12328-021-01508-1.

5. Strege RJ, Saeger W, Linke RP. Diagnosis and immunohistochemical classification of systemic amyloidoses. Report of 43 cases in an unselected autopsy series. Virchows Arch. 1998;433(1):19-27. doi: 10.1007/s004280050211.

6. Verine J, Mourad N, Desseaux K, Vanhille P, Noël LH, Beaufils H, et al. Clinical and histological characteristics of renal AA amyloidosis: a retrospective study of 68 cases with a special interest to amyloid-associated inflammatory response. Hum Pathol. 2007;38(12):1798-809. doi: 10.1016/j.humpath.2007.04.013.

7. Patel D, Agarwal R, Dhooria S, Hedge U, Patel H, Singh Sehgal I. Amyloidosis secondary to chronic pulmonary aspergillosis: Case report and a systematic review of literature. J Mycol Med. 2019;29(4):372-4. doi: 10.1016/j.mycmed.2019.100898.

8. Saha A, Theis JD, Vrana JA, Dubey NK, Batra VV, Sethi S. AA amyloidosis associated with hepatitis B. Nephrol Dial Transplant. 2011;26(7):2407-12. doi: 10.1093/ndt/gfr224.

9. Breillat P, Pourcher V, Deshayes S, Buob D, Cez A, Michel PA, et al. AA Amyloidosis in the Course of HIV Infection: A Report of 19 Cases Including 4 New French Cases and a Comprehensive Review of Literature. Nephron. 2021;145(6):675-83. doi: 10.1159/000516982.

10. Singh G, Kumari N, Aggarwal A, Krishnani N, Misra R. Prevalence of subclinical amyloidosis in ankylosing spondylitis. J Rheumatol. 2007;34(2):371-3.

11. Bektaş M, Koca N, Ince B, Yalçınkaya Y, Esen BA, Öcal ML, et al. Course and Prognosis of AA Amyloidosis in Patients with Psoriatic Arthritis: Report of Three Cases from a Single Center Cohort and Review of the Literature. Mediterr J Rheumatol. 2022;33(2):185-95. doi: 10.31138/mjr.33.2.185.

12. Bilginer Y, Akpolat T, Ozen S. Renal amyloidosis in children. Pediatr Nephrol. 2011;26(8):1215-27. doi: 10.1007/s00467-011-1797-x.

13. Delplanque M, Pouchot J, Ducharme-Bénard S, Fautrel BJ, Benyamine A, Daniel L, et al. AA amyloidosis secondary to adult onset Still's disease: About 19 cases. Semin Arthritis Rheum. 2020;50(1):156-65. doi: 10.1016/j.semarthrit.2019.08.005.

14. Ter Borg EJ, Wegewijs MA, de Bruin P. Gout and AA Amyloidosis: A Case Report and Review of the Literature. J Clin Rheumatol. 2017;23(4):233-4. doi: 10.1097/rhu.0000000000000518.

15. Escribá A, Morales E, Albizúa E, Herrero JC, Ortuño T, Carreño A, et al. Secondary (AA-type) amyloidosis in patients with polymyalgia rheumatica. Am J Kidney Dis. 2000;35(1):137-40. doi: 10.1016/s0272-6386(00)70312-x.

16. Kos I, Stilgenbauer S, Bewarder M. Renal AA amyloidosis leading to early diagnosis and treatment of takayasu arteritis: a case report and review of the literature. Clin Res Cardiol. 2020;109(11):1438-41. doi: 10.1007/s00392-020-01655-4.

17. Ostrovršnik J, Hočevar A, Lestan B, Sodin Šemrl S, Lakota K, Tomšič M. Long-term follow-up on tocilizumab treatment of AA amyloidosis secondary to polyarteritis nodosa. Amyloid. 2016;23(4):260-1. doi: 10.1080/13506129.2016.1232648.

18. Dilşen N, Koniçe M, Aral O, Erbengi T, Uysal V, Koçak N, et al. Behçet's disease associated with amyloidosis in Turkey and in the world. Ann Rheum Dis. 1988;47(2):157-63. doi: 10.1136/ard.47.2.157.

19. Bektas M, Koca N, Oguz E, Sari S, Dagci G, Ince B, et al. Characteristics and course of patients with AA amyloidosis: single centre experience with 174 patients from Turkey. Rheumatology (Oxford). 2024;63(2):319-28. doi: 10.1093/rheumatology/kead465.

20. Aktas Yilmaz B, Düzgün N, Mete T, Yazicioglu L, Sayki M, Ensari A, et al. AA amyloidosis associated with systemic lupus erythematosus: impact on clinical course and outcome. Rheumatol Int. 2008;28(4):367-70. doi: 10.1007/s00296-007-0431-5.

21. Kimura H, Komatsuda A, Sawada K, Mimori A, Baba S, Minota S. Rapidly progressed secondary amyloidosis in a patient with mixed connective tissue disease. Intern Med. 2004;43(9):878-82. doi: 10.2169/internalmedicine.43.878.

22. Ooms V, Decupere M, Lerut E, Vanrenterghem Y, Kuypers DR. Secondary renal amyloidosis due to long-standing tubulointerstitial nephritis in a patient with Sjögren syndrome. Am J Kidney Dis. 2005;46(5):e75-80. doi: 10.1053/j.ajkd.2005.07.019.

23. Sattianayagam PT, Gillmore JD, Pinney JH, Gibbs SD, Wechalekar AD, Gilbertson JA, et al. Inflammatory bowel disease and systemic AA amyloidosis. Dig Dis Sci. 2013;58(6):1689-97. doi: 10.1007/s10620-012-2549-x.

24. Kridin K, Amber KT, Comaneshter D, Cohen AD. Amyloidosis in hidradenitis suppurativa: a cross-sectional study and review of the literature. Clin Exp Dermatol. 2020;45(5):565-71. doi: 10.1111/ced.14186.

25. Pınarbaşı AS, Dursun I, Daldaban B, Günay N, Çiçek S, Şahin N, et al. Epidermolysis bullosa complicated with nephrotic syndrome due to AA amyloidosis: A case report and brief review of literature. Saudi J Kidney Dis Transpl. 2019;30(6):1450-6. doi: 10.4103/1319-2442.275492.

26. Ben Abdelghani K, Mahfoudhi M, Hriz A, El Kossai I, Khefifi A, Turki S, et al. [AA amyloidosis complicating sarcoidosis: two cases and literature review]. Rev Med Interne. 2010;31(5):369-71. doi: 10.1016/j.revmed.2009.11.009.

27. Karim F, Clahsen-van Groningen M, van Laar JA. AA Amyloidosis and IgG4-Related Disease. N Engl J Med. 2017;376(6):599-600. doi: 10.1056/NEJMc1614275.

28. Valentin R, Gürtler KF, Schaker A. Renal amyloidosis and renal failure--a novel complication of the SAPHO syndrome. Nephrol Dial Transplant. 1997;12(11):2420-3. doi: 10.1093/ndt/12.11.2420.

29. Delplanque M, Galicier L, Oziol E, Ducharme-Bénard S, Oksenhendler E, Buob D, et al. AA Amyloidosis Secondary to Primary Immune Deficiency: About 40 Cases Including 2 New French Cases and a Systematic Literature Review. J Allergy Clin Immunol Pract. 2021;9(2):745-52.e1. doi: 10.1016/j.jaip.2020.09.023.

30. Lee H, Han KH, Jung YH, Kang HG, Moon KC, Ha IS, et al. A case of systemic amyloidosis associated with cyclic neutropenia. Pediatr Nephrol. 2011;26(4):625-9. doi: 10.1007/s00467-010-1715-7.

31. Shaulov A, Prus D, Lavie D, Elias S. Duodenal amyloid A depositions in a patient with refractory Hodgkin's lymphoma: an old complication in the modern treatment era. Amyloid. 2018;25(4):263-4. doi: 10.1080/13506129.2018.1517737.

32. Linder J, Silberman HR, Croker BP. Amyloidosis complicating hairy cell leukemia. Am J Clin Pathol. 1982;78(6):864-7. doi: 10.1093/ajcp/78.6.864.

33. Terré A, Colombat M, Cez A, Martin C, Diet C, Brechignac S, et al. AA amyloidosis complicating monoclonal gammopathies, an unusual feature validating the concept of "monoclonal gammopathy of inflammatory significance"? Int J Clin Pract. 2021;75(11):e14817. doi: 10.1111/ijcp.14817.

34. Bharati J, Lahoud OB, Jhaveri KD, Izzedine H. AA amyloidosis associated with cancers. Nephrol Dial Transplant. 2023;38(6):1366-74. doi: 10.1093/ndt/gfac217.
